# Prevalence of Different Hepatobiliary Tree Variants on Magnetic Resonance Cholangiopancreatography in Patients Visiting a Tertiary Care Teaching Hospital in Karachi

**DOI:** 10.7759/cureus.12329

**Published:** 2020-12-27

**Authors:** Muhammad Qasim Naeem, Muhammad Saad Ahmed, Kamran Hamid, Muhammad Kashif Shazlee, Farheen Qureshi, Muhammad Asad Ullah

**Affiliations:** 1 Diagnostic Radiology, Dr. Ziauddin Hospital, Karachi, PAK

**Keywords:** biliary tree, hepatobiliary anatomy, biliary tree variants, mrcp, liver transplant, hepatobiliary surgeries

## Abstract

Introduction

Hepatobiliary tree variant anatomy is crucial to understand the preoperative planning of hepatobiliary surgeries. Although the presence of variant anatomy is not an absolute contraindication for liver transplantation, inadvertent mapping can lead to postoperative biliary complications. These variants are also important to be recognized in various hepatobiliary surgeries and interventional procedures. Magnetic resonance cholangiopancreatography (MRCP) is an excellent non-invasive imaging tool that can identify biliary anatomy. The purpose of the current study is focused on determining anatomical variants of the biliary tree on MRCP in our population visiting a teaching hospital in Karachi.

Methods

This cross-sectional study was conducted on patients referred to Dr. Ziauddin Hospital for MRCP. MRCP was performed on MAGNETOM Avanto, SIEMENS, Belgium, Germany. Images were analyzed on a workstation by two radiologists and a postgraduate trainee. A senior radiologist reviewed equivocal cases. SPSS 22.0 (SPSS Inc., Chicago, IL) was used for statistical analysis. Chi-square test was used to see the link between anatomical variants of biliary tree and gender. P-value of ≤0.05 was considered as statistically significant.

Results

We recruited 369 patients undergoing MRCP consecutively for our study. Out of 369, 342 patients were eligible for analysis (139 males and 203 females). Standard anatomy was found to be prevalent in 65.8%. Type 3 was the leading variant. A statistically significant difference was recorded for the type 2 anatomic variant which was more frequent in males than females (p-value <0.001), while types 1, 3, and 4 anatomic variants were found to be more in females than males but this difference was not statistically significant. Few other variants were also recorded.

Conclusion

This study is robust evidence regarding biliary variants in Pakistan. It is important to consider these variants in our region, owing to an increased trend of liver transplants and other hepatobiliary procedures.

## Introduction

The hepatobiliary tree is responsible for the drainage of bile. It consists of an intra and extrahepatic biliary system. The intrahepatic biliary system comprises of right and left main hepatic duct. Normally, the right hepatic duct has anterior and posterior hepatic ducts (RAHD and RPHD) which drain segments V, VIII and segments VI, VII, respectively [1]. The left hepatic duct is formed from branches of segments II, III and IV [1]. The right and left main hepatic ducts join at the confluence to form the common hepatic duct (CHD). By merging with the cystic duct, CHD becomes a common bile duct (CBD), which drains into the second part of the duodenum at the ampulla of the Vater.

Studies suggest that the aforementioned classical anatomy is observed in 55% to 62% of the population [1-3], and there is diversity in the anatomy of the biliary tree mentioned in the literature. Among the variants, the most common is the right posterior hepatic duct (RPHD) joining the left hepatic duct (LHD) [2,3]. Other different variants that commonly exist include, trifurcation of the CHD, RPHD joining the CHD and RPHD joining the cystic duct [1-6]. There are additionally other variations which are very rare.

With the expanding pattern in interventional radiological methods and surgical techniques, such as image-guided interventional biliary drainage procedures, laparoscopic cholecystectomies, tumor surgery and liver resection alongside liver transplantation, precise information of these anatomic variations is of considerable clinical pertinence [2,4,5,7,8], as perioperative biliary complications comprise one of the most frequently encountered reasons for morbidity and mortality [1,2,9,10].

Various techniques are available for imaging of the biliary tree such as intravenous cholangiography, drip-infusion CT cholangiography, endoscopic retrograde cholangiopancreatography (ERCP), intraoperative cholangiography and magnetic resonance cholangiopancreatography (MRCP). Intravenous cholangiography does not provide a detailed visualization of the biliary tree [4]. ERCP and intraoperative cholangiography are highly accurate but invasive [4]. MRCP is an excellent non‑invasive imaging technique for the visualization of detailed biliary anatomy [4].

Many studies have been conducted to estimate the prevalence of different biliary tree variants as described by Nayman et al. [2], Pesce et al. [3], Uysal et al. [6], and Gursoy et al. [7], but so far local data remain scarce. The objective of this study is to determine the prevalence of anatomical variants of the biliary tree in our population using the MRCP technique at a tertiary care hospital.

## Materials and methods

Methodology

This cross-sectional study was conducted from January 2019 to July 2020 in the Radiology Department at Ziauddin Hospital, Karachi. After informed consent, all patients of age more than 18 years undergoing MRCP were consecutively sampled irrespective of gender. A sample size of 369 patients with MRCP was calculated by WHO sample size calculator using 60% prevalence of normal biliary anatomy reported in the literature and keeping a margin of error at 5%. Patients with prior hepatic resection, benign or malignant neoplastic lesions, severe biliary tree obstruction, motion artefacts and poor quality images were excluded from the study.

We adopted the updated biliary tree classification system as mentioned by Nayman et al. [2]. This classification is based upon Yoshida’s classification [2].

Image acquisition

Patients were requested to fast for four hours and were given pineapple juice 15 minutes before the study. MRCP was performed by using 1.5T MRI MAGNETOM Avanto, SIEMENS, Belgium, Germany, using a body coil. The examination was performed in the supine position. Our institutional protocol included: axial and coronal thin slab single-shot turbo spin-echo (HASTE) sequences (TR:1350 ms, TE:92ms, FOV 256 × 205, 4.0 mm), axial and coronal thin slab fat-suppressed single-shot turbo spin-echo (HASTE) sequences (TR:1900 ms, TE 108 ms, FOV 384 × 288, 4.0 mm), axial and coronal thin slab Gradient echo Fast imaging with steady-state precession (TRUFI) sequences (TR:3.95ms, TE:1.71 ms, FOV 256 × 230, 4.0 mm), respiratory-triggered oblique coronal 3D turbo spine-echo (SPACE) sequence (TR:3162 ms TE:698 ms, FOV 384 × 354, 1.0 mm), and respiratory-triggered thick slab Single-shot turbo spine-echo (HASTE) sequence (TR:4500 ms, TE:756ms, FOV 384 × 269, 50 mm).

Image analysis

Images were analyzed on a workstation. Each scan was interpreted by two radiologists; each having five years of experience and a postgraduate resident with four years’ experience. A senior radiologist with 15 years of experience in body imaging further interpreted the images in equivocal­ cases. Data for different biliary tree variants with demographic details were recorded. SPSS 22.0 was used for statistical analysis. Chi-square test was applied to see the differences between the anatomic variations of the biliary tree among males and females. P-value of ≤0.05 was considered as statistically significant.

## Results

A total of 369 patients undergoing MRCP were included in the study. Out of these, 27 patients (7.3%) were excluded from the study with the majority due to poor quality images or motion artefacts (n=13), biliary tree obstruction obscuring anatomical details (n=8), cirrhosis (n=4), neoplastic lesion (n=1) and hepatic resection (n=1). Thus, a total of 342 patients were finally included in the study, 139 males (40.6%) and 203 females (59.4%), respectively. The mean age of the patients was 48.5±16.7.

Table 1 shows the frequency of the biliary tree variation. Conventional anatomy (type 1) was found in most of the patients. i.e., 225 (65.8%). The next most common variant was RPHD joining the LHD (Type 3) in 49 subjects (14.3 %), trifurcation of the biliary tree (Type 2) in 36 subjects (10.5%) and RPHD joining the CHD (Type 4) in 22 patients (6.4%). Other less commonly encountered variants were also observed, type 5 in two patients (0.6%), type 9 in three patients (0.9%), types 10 and 11 in one patient (0.3%) each and type 14 in three patients (0.9%). Standard anatomy was higher in female patients (67.0%) than in male patients (64.0%). Among variants, types 3 and 4 were also recorded higher in female patients, i.e., 16.3% and 7.4% respectively, while type 2 was found to be more prevalent in males 18.7%.

**Table 1:** Frequency of anatomical variants of the biliary tree on MRCP within gender in a sample of Pakistani population visiting a tertiary care hospital (n = 342) MRCP: magnetic resonance cholangiopancreatography.

Variation	Frequency (n)	Percentage (%)	Gender	
			Male	Female
Type 1	225	65.8	89 (64.0%)	136 (67.0%)
Type 2	36	10.5	26 (18.7%)	10 (4.9%)
Type 3	49	14.3	16 (11.5%)	33 (16.3%)
Type 4	22	6.4	7 (5.0%)	15 (7.4%)
Type 5	2	0.6	0 (0.0%)	2 (1.0%)
Type 9	3	0.9	1 (0.7%)	2 (1.0%)
Type 10	1	0.3	0 (0.0%)	1 (0.5%)
Type 11	1	0.3	0 (0.0%)	1 (0.5%)
Type 14	3	0.9	0 (0.0%)	3 (1.5%)
			100.0%	100.0%
Total	342	100.0	139 (40.6%)	203 (59.4%)

**Figure 1:**
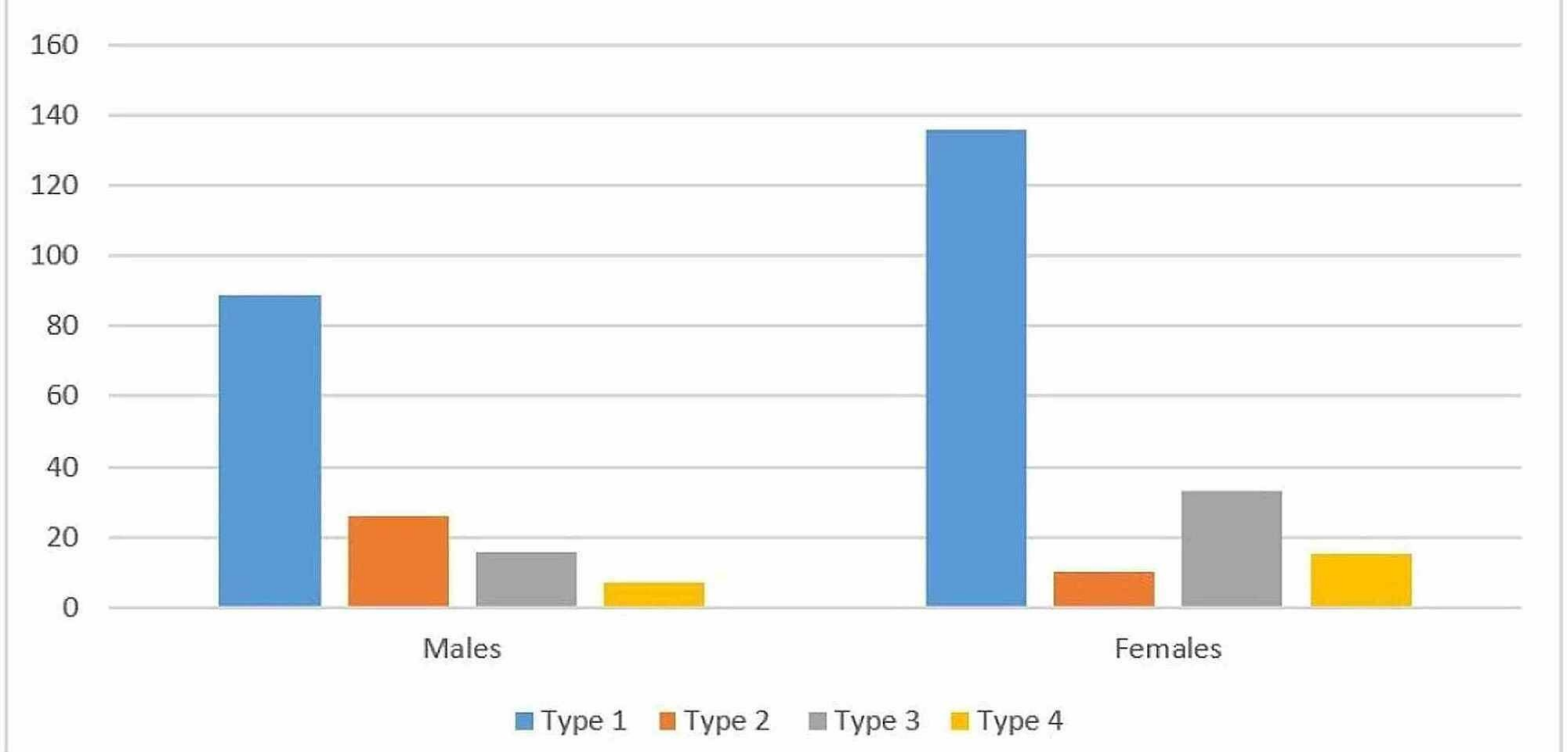
Graphical representation of the four most common anatomic variants recorded in our study (n=332)

Figure 1 shows the graphical representation of most commonly encountered variants (n=332) within gender. To compare the anatomical variations between females and males, we used the chi-square test of proportion (Table 2). We had enough data available for comparison of types 1-4 categories and the statistical comparison was performed on the same. There was a statistically significant difference in the proportion of type 2 anatomical variant; as it was more common among males as compared to females (18.7% v/s 4.9%; p-value <0.001). For the rest of the types, we presented the comparison of frequency only (Table 1); no statistical significance could be calculated due to not having enough samples in each of the categories.

**Table 2:** Comparison of most common anatomical variations of the biliary tree among males and females in the Pakistani population (n=332)

Anatomical variations		Gender		p-Value
		Male	Female	
Type 1	n	89	136	0.570
	%	64%	67%	
Type 2	n	26	10	<0.001
	%	18.7%	4.9%	
Type 3	n	16	33	0.219
	%	11.5%	16.3%	
Type 4	n	7	15	0.384
	%	5.0%	7.4%	

## Discussion

In recent years, the number of hepatobiliary operations, interventional radiological techniques, extensive or partial hepatectomy/segmentectomy and liver transplants in our region has increased. Therefore, an in-depth study of intrahepatic biliary anatomy is of immense importance where inadvertent mapping due to variant anatomy may lead to intra and peri-operative complications.

With recent advancements in imaging techniques, mapping of hepatobiliary anatomy can be easily performed. Preoperative mapping with ERCP can be performed, but it is an invasive procedure [4]. Another invasive technique is intraoperative cholangiography that can only be performed during surgery. Drip-infusion CT cholangiography requires contrast media and is now rarely performed because of the limited availability of contrast media and possible contrast-induced adverse reactions [11]. MRCP is an invaluable tool that can easily identify the variations of the hepatobiliary tree. MRCP is a non-invasive and safe technique with no ionizing radiation and procedure-related complications. In addition to the assessment of the biliary system, it can also assess parenchymal abnormalities of the liver and pancreas. In post-transplant patients, MRCP has also been the best non-invasive modality for diagnosing postoperative biliary problems [12].

Our results demonstrated normal biliary anatomy (Yoshida type 1) in 65.8% of subjects which is slightly higher when compared to prior studies with deviation from normal anatomy observed in 34.2% of subjects. European studies such as Pesce et al. [3] and Nayman et al. [2] recorded 55% and 62% of conventional anatomy in their study, respectively, while Cucchetti et al. [13] and Mariolis-Sapsakos et al. [9] reported a similar ratio in their studies, i.e., 64.5% and 65%, respectively. The frequency of type 2 (trifurcation formed by RAHD, RPHD and LHD) and type 3 (RPHD draining into LHD) variations were equivocal in prior studies [2,3,6]. Our study demonstrated type 3 anomaly as the most frequently encountered anatomical variation of the biliary tree which is in concordance with Nayman et al. [2], Pesce et al. [3] and Gihan [14] followed by trifurcation variant (Type 2). Previous studies have also shown that RPHD joining the common hepatic duct is the fourth most encountered variant similar to our study [2,3,13]. The types 1, 3 and 4 anatomic variants were recorded more frequently in females than in males while type 2 was more prevalent in males as compared to females. We found statistical significance among gender in type 2 variation, as it was more prevalent among males (18.7% v/s 4.9%; p-value <0.001). Biliary tree variants have been linked to gender, Cucchetti et al. [13] reported variant pattern was more recorded in females than in males (45% vs 26%; p-value=0.005), however, in Asian studies [8,15] no statistically significant link between gender and variant anatomy was recorded.

**Figure 2:**
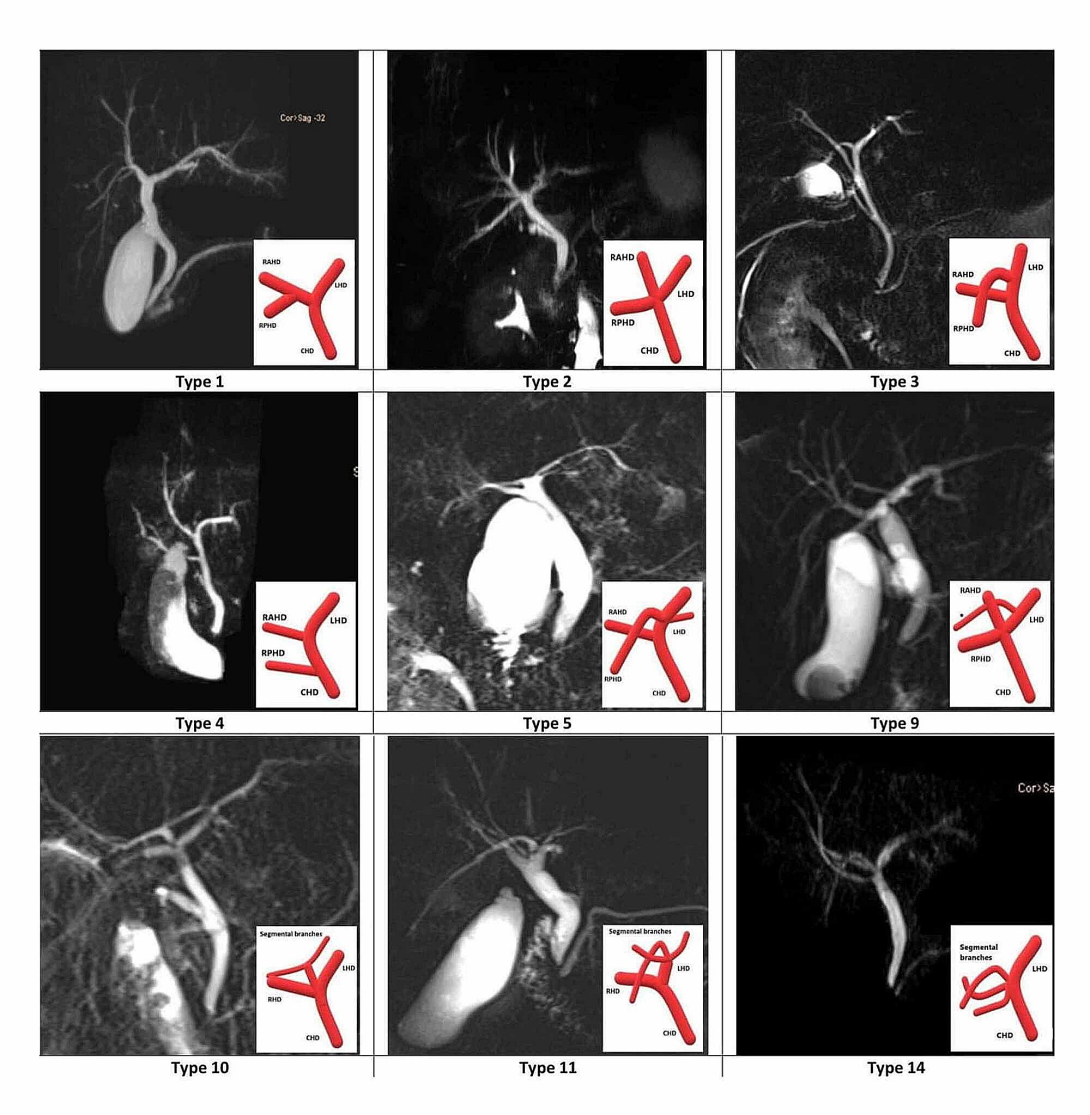
MRCP images demonstrating anatomical variants of biliary tree recorded in our study *Segmental branch. MRCP: magnetic resonance cholangiopancreatography.

We recorded few other variations, i.e., type 5 variation (RPHD draining in LHD with a segmental duct draining at the same site) in two patients, type 9 in three patients (trifurcation associated with a right segmental duct draining in LHD), type 10 in one patient (a segmental duct from right and left lobes forming a confluence which drains to the CHD), type 11 in one patient (three segmental branches draining in LHD forming a trifurcation) and type 14 in three patients (three segmental ducts forming trifurcation on the right side; Figure 2).

Several other anatomical variants have also been reported in the literature; however, these are uncommonly seen and clinically significant data are scarce. Examples include isolated segmental ducts draining to right or left hepatic duct, isolated segmental branches draining to CHD or cystic duct as well as complex biliary variants [1-3,6,9,16]. In addition, variant biliary tree systems often linked to variation in the portal vein and their assessment are important to reduce the risk of iatrogenic insults [4,17].

Related to the development of the hepatobiliary system, the organogenesis starts in the fourth week by emerging as an outpouching from the distal part of the foregut known as the hepatic diverticulum. The hepatic diverticulum amplifies quickly and isolates into two sections, the cranial part which is the primordium for the liver and intrahepatic biliary tree; and the caudal part which turns into the primordium of the gallbladder with its stalk framing the cystic duct. The tail of the diverticulum interfacing the hepatic and cystic duct to the duodenum turns into the common bile duct. The portion above the intersection of the cystic duct with CBD is the CHD. Intrahepatic biliary channels arise from the ductal plate which itself is a twofold layer of cells encircling the portal vein [18,19]. The pathway of divarication of portal vein decides the intrahepatic biliary branching pattern. This also shows that variants of biliary channels follow the tract of aberrant portal venous branches [10,19,20].

Biliary complications occur frequently in living donor liver transplant (LDLT). Biliary reconstruction is technically challenging and variant anatomy leads to increase postoperative biliary complications and leakage. Detailed knowledge of the biliary anatomy of a donor is an important step in the planning process of the right liver lobe transplant to save the hepatectomy of a donor and reduce postoperative complications of the recipient. Biliary complications are also the most frequent cause of donor morbidity, arising after LDLT in 7-10% of donors [21].

While liver transplantation is not impeded by hepatobiliary tree variations, lack of understanding of these variations may lead to inadvertent ligation of major segmental biliary ducts that can lead to graft failure or cirrhosis [2,17]. Varotti et al. [10] and Nakamura et al. [20] concluded in their studies that no absolute contraindication for transplantation with biliary system variations. However, a precise preoperative assessment of the biliary system is crucial for effective transplantation planning. For example, during left hepatectomy in a patient with type 3 variation according to Yoshida classification, RPHD can be ligated resulting in cirrhosis of segments 6 and 7 [2]. In the planning of left hepatectomy, biliary anatomy is necessary to be mapped as ligation of aberrant RPHD or RAHD can result in biliary cirrhosis of segments 6/7 and 5/8, respectively. In the presence of type 2 or 3 pattern, safe donation of the right lobe, or both lobes cannot be performed, respectively [11,22]. Other postoperative complications include biliary leaks or biloma, biliary stenosis, biliary peritonitis, non-anastomotic strictures secondary to vascular insufficiency and biliary cast syndrome, etc. [21,23].

Laparoscopic surgery is now the standard operation for performing cholecystectomy. Although bile duct injury is lower than 1% bile duct injuries are the most grievous in laparoscopic cholecystectomy, accounting for 0.3-0.7%, and are a significant factor in patient’s morbidity [1,11,24,25]. It is also crucial to pay attention to Yoshida type 4 variation in which aberrant RPHD joining the CHD may get injured during surgery. Furthermore, recognition of accessory hepatic ducts such as segmental branches joining the cystic duct [2] and the duct of Luschka (chole-cystohepatic duct) is also important to be mapped to avoid unnecessary postoperative biliary complications.

## Conclusions

This study is robust evidence regarding the prevalence of biliary tree anatomic variants in Pakistan, reporting that conventional anatomy is the most common biliary pattern in our population. Type 3 was the most commonly encountered variant followed by types 2 and 4 overall. Type 2 anatomic variants were found to be more frequent in males than females (p-value <0.001), while types 1, 3 and 4 anatomic variants were found to be more in females than males but this difference was not statistically significant. Knowledge of these anatomical variations is useful to prevent iatrogenic injuries which increase postoperative morbidity and mortality.

## References

[1] Mortelé KJ, Ros PR (2001). Anatomic variants of the biliary tree: MR cholangiographic findings and clinical applications. AJR Am J Roentgenol.

[2] Nayman A, Özbek O, Erol S, Karakuş H, Kaya HE (2016). Magnetic resonance cholangiopancreatography evaluation of intrahepatic bile duct variations with updated classification. Diagn Interv Radiol.

[3] Pesce A, Ultimo LE, Piccoli M (2020). Anatomic variations of intrahepatic biliary system at magnetic resonance cholangio-pancreatography: a single institution experience and a systematic review of the literature. EMBJ.

[4] Sureka B, Bansal K, Patidar Y, Arora A (2016). Magnetic resonance cholangiographic evaluation of intrahepatic and extrahepatic bile duct variations. Indian J Radiol Imaging.

[5] Choi JW, Kim TK, Kim KW, Kim AY, Kim PN, Ha HK, Lee MG (2003). Anatomic variation in intrahepatic bile ducts: an analysis of intraoperative cholangiograms in 300 consecutive donors for living donor liver transplantation. Korean J Radiol.

[6] Uysal F, Obuz F, Uçar A, Seçil M, Igci E, Dicle O (2014). Anatomic variations of the intrahepatic bile ducts: analysis of magnetic resonance cholangiopancreatography in 1011 consecutive patients. Digestion.

[7] Gürsoy Çoruh A, Gülpınar B, Baş H, Erden A (2018). Frequency of bile duct confluence variations in subjects with pancreas divisum: an analysis of MRCP findings. Diagn Interv Radiol.

[8] Taghavi SA, Niknam R, Alavi SE, Ejtehadi F, Sivandzadeh GR, Eshraghian A (2017). Anatomical variations of the biliary tree found with endoscopic retrograde cholagiopancreatography in a referral center in Southern Iran. Middle East J Dig Dis.

[9] Mariolis-Sapsakos T, Kalles V, Papatheodorou K (2012). Anatomic variations of the right hepatic duct: results and surgical implications from a cadaveric study. Anat Res Int.

[10] Varotti G, Gondolesi GE, Goldman J (2004 Apr). Anatomic variations in right liver living donors. J Am Coll Surg.

[11] Hyodo T, Kumano S, Kushihata F (2012). CT and MR cholangiography: advantages and pitfalls in perioperative evaluation of biliary tree. Br J Radiol.

[12] Novellas S, Caramella T, Fournol M, Gugenheim J, Chevallier P (2008). MR cholangiopancreatography features of the biliary tree after liver transplantation. AJR Am J Roentgenol.

[13] Cucchetti A, Peri E, Cescon M (2011). Anatomic variations of intrahepatic bile ducts in a European series and meta-analysis of the literature. J Gastrointest Surg.

[14] Gamal GH (2017). Minimizing the postoperative biliary complications in living donor liver transplantation, by utility of preoperative non-enhanced magnetic resonance cholangiopancreatography. Egypt J Radiol Nucl Med.

[15] Deka P, Islam M, Jindal D, Kumar N, Arora A, Negi SS (2014 Jan). Analysis of biliary anatomy according to different classification systems. Indian J Gastroenterol.

[16] Sarawagi R, Sundar S, Raghuvanshi S, Gupta SK, Jayaraman G (2016). Common and uncommon anatomical variants of intrahepatic bile ducts in magnetic resonance cholangiopancreatography and its clinical implication. Pol J Radiol.

[17] Hennedige T, Anil G, Madhavan K (2014). Expectations from imaging for pre-transplant evaluation of living donor liver transplantation. World J Radiol.

[18] Roskams T, Desmet V (2008). Embryology of extra- and intrahepatic bile ducts, the ductal plate. Anat Rec (Hoboken).

[19] Abou-Khalil JE, Bertens KA (2019). Embryology, anatomy, and imaging of the biliary tree. Surg Clin North Am.

[20] Nakamura T, Tanaka K, Kiuchi T (2002). Anatomical variations and surgical strategies in right lobe living donor liver transplantation: lessons from 120 cases. Transplantation.

[21] Catalano OA, Singh AH, Uppot RN, Hahn PF, Ferrone CR, Sahani DV (2008). Vascular and biliary variants in the liver: implications for liver surgery. Radiographics.

[22] Lee VS, Morgan GR, Teperman LW (2001). MR imaging as the sole preoperative imaging modality for right hepatectomy: a prospective study of living adult-to-adult liver donor candidates. AJR Am J Roentgenol.

[23] Simoes P, Kesar V, Ahmad J (2015). Spectrum of biliary complications following live donor liver transplantation. World J Hepatol.

[24] Pesce A, Diana M (2018). Critical view of safety during laparoscopic cholecystectomy: from the surgeon's eye to fluorescent vision. Surg Innov.

[25] Pesce A, Palmucci S, La Greca G, Puleo S (2019). Iatrogenic bile duct injury: impact and management challenges. Clin Exp Gastroenterol.

